# Single-Cell Transcriptomics Reveals Immune Modulation by Telmisartan in Colorectal Cancer

**DOI:** 10.3390/cells15080729

**Published:** 2026-04-20

**Authors:** Jinxin Li, Decao Yang, Xiaoyue Wang, Runqing Ju, Shaomeng Chen, Jingyi Zhao, Jiaxing Xu, Jiaxin Chen, Jiayu Ye, Baohui Xu, Qianqian Yin, Yan Wang

**Affiliations:** 1 Institute of Medical Innovation and Research, Peking University Third Hospital, Beijing 100191, China; 2110301321@bjmu.edu.cn (J.L.);; 2 Medical Research Center, Peking University Third Hospital, Beijing 100191, China; 3 Emergency Department, Peking University Third Hospital, Beijing 100191, China; 4 Department of Medicine, School of Medicine, Stanford University, Stanford, CA 94305, USA; 5 Division of Vascular Surgery, Department of Surgery, School of Medicine, Stanford University, Stanford, 94305 CA, USA

**Keywords:** telmisartan, colorectal cancer, tumor microenvironment, macrophage, T cell

## Abstract

**Highlights:**

**What are the main findings?**

- **Telmisartan downregulates pro-tumoral macrophage activity:** Single-cell transcriptomics revealed that telmisartan significantly suppresses M2-associated macrophage programs (including *Mrc1* and *Spp1* expression) and inhibits their cell proliferation-related pathways.
- **Telmisartan drives an anti-tumor T cell shift:** Treatment increased the infiltration and proportion of cytotoxic CD8^+^ T cells at the tumor border, reduced regulatory T cell counts, and enhanced MHC class I-mediated antigen presentation.

**What are the implications of the main findings?**

- **Reshaping the immunosuppressive microenvironment:** The dual action of downregulating tumor-promoting macrophages while simultaneously activating cytotoxic T cells demonstrates that telmisartan can structurally improve the tumor immune landscape.
- **Potential for clinical repurposing:** These single-cell mechanistic insights provide a strong rationale for investigating safe, widely available, and cost-effective angiotensin receptor blockers like telmisartan as adjuvants to boost the efficacy of cancer immunotherapies.

**Abstract:**

Telmisartan, an angiotensin II type 1 receptor blocker with established anti-inflammatory and antihypertensive properties, has been reported to inhibit tumor cell proliferation, yet its impact on the tumor immune microenvironment remains poorly understood. In this study, we evaluated the immunomodulatory effects of telmisartan using a syngeneic MC38 colorectal cancer model in C57BL/6 mice. Daily intragastric administration of telmisartan significantly suppressed tumor growth and reduced endpoint tumor weight compared with controls. To elucidate the underlying mechanisms, we performed single-cell RNA sequencing on tumor-infiltrating CD45^+^ immune cells and revealed a macrophage-dominated immune landscape comprising multiple transcriptionally distinct subclusters. Telmisartan broadly downregulated pro-tumoral and M2-associated macrophage programs, including decreased expression of genes such as *Mrc1* and *Spp1*, while also suppressing cell proliferation-related pathways. In contrast to its overall suppressive impact on macrophages, telmisartan increased the proportion of cytotoxic CD8^+^ T cells, reduced regulatory T cell counts, and enhanced major histocompatibility complex class I antigen presentation, consistent with an immune-activating effect. These results indicate that telmisartan reshapes the colorectal tumor immune microenvironment by simultaneously attenuating tumor-promoting macrophage activity and augmenting cytotoxic T cell responses. Overall, this study provides a single-cell framework to understand how angiotensin receptor blockade reshapes tumor-infiltrating immune programs, highlighting the translational potential of repurposing telmisartan for novel cancer immunotherapy strategies.

## 1. Introduction

Colorectal cancer (CRC) remains a global health challenge, ranking as the third most common malignancy and the second leading cause of cancer-related mortality worldwide [1]. Current clinical strategies, including surgery, radiotherapy, chemotherapy, and emerging technologies such as immunotherapy and targeted therapy, have significantly advanced patient care [2,3,4,5]. Despite this progress, overall prognosis remains highly dependent on stage at diagnosis. For patients with distant metastases, 5-year survival rates are below 20% in both the United States and Japan [6,7]. Moreover, classical chemotherapy regimens are frequently associated with common adverse effects, such as paresthesia and neutropenia, and the associated costs often exceed $10,000 [8,9]. Therefore, exploring novel therapeutic agents, particularly the repurposing of widely used, safe, and inexpensive drugs to enhance efficacy while reducing side effects and costs, represents a promising prospect for advancing colorectal cancer treatment.

Angiotensin II (Ang II), the principal effector peptide of the renin–angiotensin–aldosterone system, is best known for its roles in blood pressure regulation and fluid homeostasis. Beyond these cardiovascular functions, mounting evidence implicates Ang II signaling in tumorigenesis and metastasis [10,11]. The Ang II type 1 receptor (AT1R) is expressed on multiple cancer cell types, including colorectal cancer cells [12], and activation of the Ang II-AT1R axis promotes expression of pro-angiogenic and pro-proliferative factors such as vascular endothelial growth factor, angiopoietin-2, fibroblast growth factors and platelet-derived growth factor [13].

Since the approval of losartan in 1995, AT1R blockers (ARBs) have undergone substantial development and have become cornerstone therapies in the management of hypertension [14]. In recent years, the potential antitumor effects of ARBs have garnered increasing attention, with telmisartan emerging as a representative candidate. Preclinical studies report that telmisartan inhibits growth of various tumors in vitro and in vivo [15,16,17,18], and it exerts antiproliferative and pro-apoptotic effects in colorectal cancer cell lines [19,20,21]. In addition to AT1R antagonism, telmisartan acts as a partial agonist of peroxisome proliferator-activated receptor γ (PPARγ), a nuclear receptor implicated in tumor biology and chemosensitivity [22]. Notably, some antitumor effects of telmisartan have been attributed to its PPARγ-modulating activity rather than AT1R blockade [23].

Despite extensive work on telmisartan’s direct effects on tumor cells and angiogenesis, its impact on the tumor immune microenvironment remains under-explored. Immune dysfunction contributes substantially to colorectal cancer pathogenesis: chronic intestinal inflammation elevates CRC risk and shapes a tumor-promoting microenvironment [24]. Specifically, Ang II signaling has been reported to promote cancer-associated extramedullary hematopoiesis and drive the generation of tumor-promoting macrophages [25]. Moreover, other ARBs such as valsartan and candesartan (the chemical structural formulas of telmisartan, valsartan, and candesartan are provided in Figure S1) have been shown to augment antitumor immunity mediated by T cells and macrophages and to sensitize tumors to checkpoint blockade in preclinical models [26,27]. Mechanistically, the blockade of the AT1R axis deprives tumor cells of crucial pro-angiogenic and proliferative signals, while the immunomodulatory properties of these structures simultaneously alleviate the immunosuppressive microenvironment, rationalizing their repurposing for colorectal cancer therapy.

On the basis of these observations, we hypothesized that telmisartan may exert antitumor effects in CRC in part by reshaping the tumor immune microenvironment. To test this hypothesis, we established a syngeneic murine colorectal cancer model and applied single-cell RNA sequencing (scRNA-seq) to comprehensively profile immune populations within tumors following telmisartan treatment. Our aim was to define telmisartan-induced immune changes at single-cell resolution and to identify potential immunomodulatory mechanisms, ultimately providing a strong rationale for repurposing this widely used antihypertensive drug to enhance current cancer immunotherapies.

## 2. Materials and Methods

### 2.1. Cell Lines and Cell Culture

MC38 cells were obtained from the American Type Culture Collection. The culture conditions were identical to those described in our previous study [28]. Briefly, the cells were maintained in basic Dulbecco’s Modified Eagle Medium supplemented with 10% fetal bovine serum, 1% penicillin/streptomycin, 1% Non-Essential Amino Acids, 1% sodium pyruvate, 1% 4-(2-hydroxyethyl)-1-piperazineethanesulfonic acid, and 1% glutamine. All cell lines were mycoplasma-negative and used within 10 passages.

### 2.2. Tumor Model and Telmisartan Administration

Female C57BL/6 mice (6–8 weeks) were purchased from Beijing Vital River Laboratory (Beijing, China). All animal protocols were approved by the Animal Care and Use Committee of Peking University and all mice were housed in specific-pathogen-free conditions. MC38 cells (5 × 10^5^) were implanted subcutaneously into the mice (n = 10/group) Five mice per group were allocated for scRNA-seq, and the remaining five were used for IHC. Treatment with telmisartan (5 mg/kg) or vehicle was initiated at the time of implantation and administered intragastrically daily. The dose and schedule were chosen based on previous reports [29,30]. Tumor growth was measured using calipers every 2–3 days. Tumor size was calculated as 0.5 × Length × Width^2^. Tumors were harvested from euthanized mice on the 13th day of the experiment.

### 2.3. Immunohistochemistry

Paraffin-embedded tumor sections (4 μm) were subjected to heat-induced antigen retrieval and stained using standard biotin-streptavidin-peroxidase protocols. Sections were incubated overnight at 4 °C with primary antibodies against CD8 [Cat#: GT211229, Gene Tech, Shanghai, China], CD206 [Cat#: 60143-1-lg, Proteintech, Rosemont, IL, USA], and CD31 [Cat#: 77699S, Cell Signaling, Danvers, MA, USA]. Detection was performed using biotinylated secondary antibodies followed by a DAB substrate kit [Cat#: 550880, BD Bioscience, Franklin Lakes, NJ, USA], with hematoxylin counterstaining. Positively stained cells were quantified in five randomly selected high-power fields per section using the National Institutes of Health’s ImageJ software (version 1.49, https://imagej.net/ij/). The tumor border was defined as the region adjacent to the margin of the tumor tissue, while the tumor core represented the central region of the tumor mass.

### 2.4. Flow Cytometry

Subcutaneous tumor tissues were harvested and isolated into single cells using a tumor dissociation kit for mouse tissues (Cat#: 130-096-730, Miltenyi, Bergisch Gladbach, Germany). The resulting single-cell suspension was washed with PBS and stained with Calcein AM (Cat#: 564601, BD Bioscience) and anti-CD45 antibodies [Cat#: 103106, Biolegend, San Diego, CA, USA]. Live CD45^+^ immune cells were isolated by FACS.

### 2.5. Single-Cell RNA Sequencing

Isolated CD45^+^ immune cells from five mice in each group were pooled for scRNA-seq. To distinguish between the drug-treated and control groups, sorted cells from each group were labelled with distinct BD Mouse Immune Single-Cell Multiplexing Sample Tags (Cat#: 633793, BD Bioscience) according to the manufacturer’s instructions. After washing, the labelled cells were pooled together for subsequent capture. Single-cell libraries were constructed using the BD Rhapsody™ Single-Cell Analysis System (BD Biosciences) following the manufacturer’s instructions. Briefly, the pooled single-cell suspension was loaded into a BD Rhapsody™ Cartridge (Cat#: 633733, BD Bioscience) to capture single cells with Cell Capture Beads in microwells. Cells were lysed, and poly-A mRNA, along with Sample Tag oligos, were captured onto the beads via hybridization. The beads were then retrieved magnetically, and cDNA was synthesized via reverse transcription. Following Exonuclease I treatment to remove unbound oligos, the library preparation was divided into two workflows: (1) For Whole Transcriptome Analysis, the cDNA underwent Random Priming and Extension followed by PCR amplification; (2) For Sample Tag library generation, the tagged sequences were specifically amplified using Sample Tag PCR primers. Both libraries were indexed using Illumina-compatible P5 and P7 sequences, purified using AMPure XP magnetic beads, and quantified using a Qubit Fluorometer prior to sequencing. The resulting libraries were sequenced on the NovaSeq 6000 (Illumina, San Diego, CA, USA) with a paired-end read length of 2 × 150 bp, targeting approximately 20,000 reads per cell for the WTA library and 600 reads for the Sample Tag library. Raw sequencing data were processed using the BD Rhapsody™ WTA analysis pipeline (version 1.11) for cell barcode and UMI counting, followed by standard quality control and downstream analyses using Seurat.

### 2.6. ScRNA-Seq Analysis

ScRNA-seq data were processed via the “Seurat” package (version 5.3.0) in R (version 4.5.1). Low-quality cells were excluded on the basis of the following criteria: feature counts < 150 or >5000, and percent mitochondrial read >20%. Data normalization was performed via the “NormalizeData” function, and scaling was achieved via the “ScaleData” function. Doublets were identified and removed via the “DoubletFinder” package (version 2.0.4). Dimensionality reduction was performed using uniform manifold approximation and projection (UMAP) for visualization, and clustering was performed using Seurat’s “FindNeighbors” function; the optimal clustering resolution was selected with the “clustree” package (version 0.5.1). Due to the confounding effects of abundant macrophage infiltration, which led to the ubiquitous expression of macrophage markers across all clusters, we adopted the following strategy: non-macrophage populations were identified first using canonical marker genes: T cells (*Cd3d*, *Cd3g*, *Skap1*, and *Gimap*) [31], dendritic cells (*Flt3*, *Zbtb46*, and *Kit*) [32,33], and neutrophils (*Cxcr2*, *S100a8*, *S100a9*, and *Csf3r*) [34] (Figure S2). Other common immune populations, including B cells (*Cd79a* and *Cd19*) and Natural Killer cells (*Klrb1c* and *Ncr1*) were also screened, but the expression of these markers was essentially negligible in our dataset (Figure S2). Cells not assigned to these populations were classified as macrophages based on high expression of *Adgre1*, *Cd86*, and/or *Mrc1* [34]. To elucidate functional characteristics of these cell populations, GO analysis and GSEA were performed via the “GSEABase” package (version 1.70.0) and “clusterProfiler” package (version 4.16.0). Cell–cell communication analysis was accomplished using the “CellChat” package (version 2.2.0). Trajectory analysis was performed via the “monocle3” package (version 1.4.26).

### 2.7. Statistical Analysis

SPSS software 27.0.1 (IBM, Chicago, IL, USA) and GraphPad Prism 9.3 (GraphPad Software, San Diego, CA, USA) were used to conduct the statistical analyses and visualization. Data are presented as mean ± SEM. Student’s *t*-test was used for two-group comparisons, and two-way repeated measures ANOVA was used for two-factor models. *p* < 0.05 was considered statistically significant.

## 3. Results

### 3.1. Telmisartan Inhibits the Growth of MC38 Colorectal Tumors In Vivo

**Figure 1:**
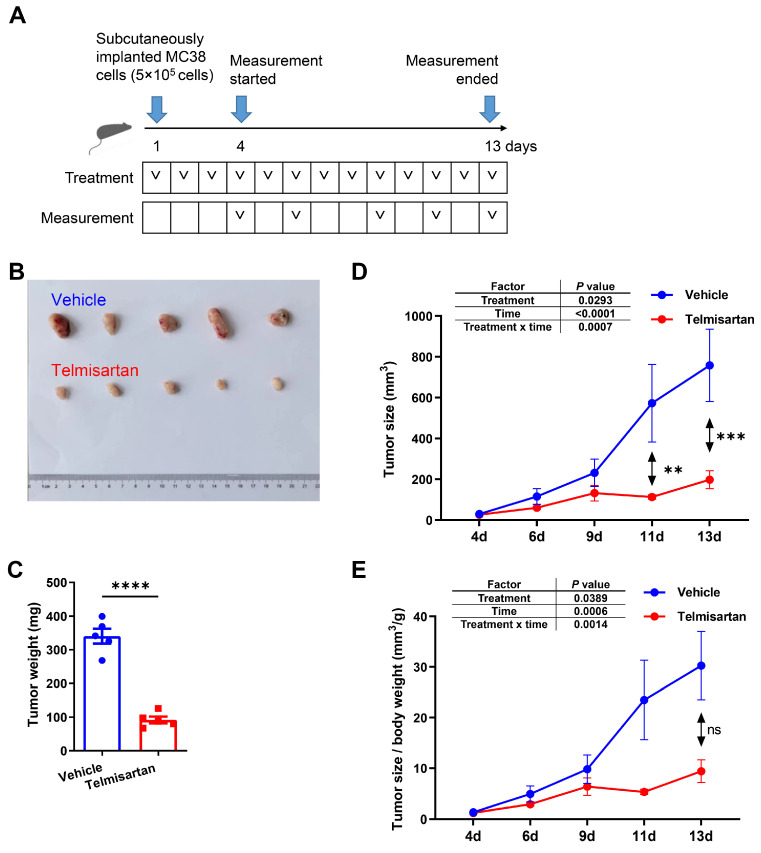
Telmisartan inhibits the growth of MC38 colorectal tumors in vivo (n = 10/group). (**A**) Schematic diagram of the treatment and measurement procedure. C57BL/6 mice were subcutaneously implanted with MC38 cells and received either vehicle or telmisartan intragastrically (i.g.) daily. Measurements were implemented on days 4, 6, 9, 11, 13 and the whole experiment ended on day 13. (**B**) Photo of the harvested tumors of each group at the experimental endpoint. (**C**) Histogram showing the tumor weight of each group at the end of the experiment. A *t*-test was used. (**D**,**E**) Line graphs showing the variation trend of (**D**) tumor size and (**E**) tumor size to body weight ratio of each group during the experiment. The significance of the main effect of the factors was measured using two-way repeated measures ANOVA. Statistical differences between the vehicle and telmisartan groups at specific individual time points were determined using a post hoc Šídák’s multiple comparisons test. ** *p* < 0.01, *** *p* < 0.001, **** *p* < 0.0001. ns, not significant.

The antitumor efficacy of telmisartan was evaluated in vivo using a syngeneic murine colorectal cancer model. MC38 cells were subcutaneously implanted into C57BL/6 mice. To simulate the clinical usage of telmisartan as an oral medication, mice were randomized to receive daily intragastric (i.g.) gavage of either vehicle control or telmisartan, with measurements starting on day 4 (Figure 1A). At the experimental endpoint (day 13), tumors from the telmisartan-treated group were visibly smaller than those from the control group (Figure 1B). This was confirmed by endpoint tumor weight, which was significantly reduced in the telmisartan-treated group compared to the control group (Figure 1C). Longitudinal monitoring of tumor volume demonstrated that telmisartan significantly suppressed the tumor growth rate, with significant differences emerging at specific time points (Figure 1D). A two-way repeated measures ANOVA confirmed a significant interaction between treatment and time, indicating that the suppressive effect of telmisartan on tumor size became more pronounced over time. We also monitored body weight to assess potential systemic toxicity. Intriguingly, telmisartan treatment led to a significantly lower body weight compared to the control group (Figure S3). To account for this difference, we analyzed the tumor size to body weight ratio. Although stringent post hoc multiple comparisons at single time points did not reach statistical significance, the overall longitudinal trajectory of this ratio remained significantly lower in the telmisartan-treated group, as confirmed by the two-way ANOVA (Figure 1E). This further supports the potent anti-tumor efficacy of telmisartan independent of body weight changes.

### 3.2. Macrophages Dominate the Tumor Immune Microenvironment and Exhibit High Heterogeneity

**Figure 2:**
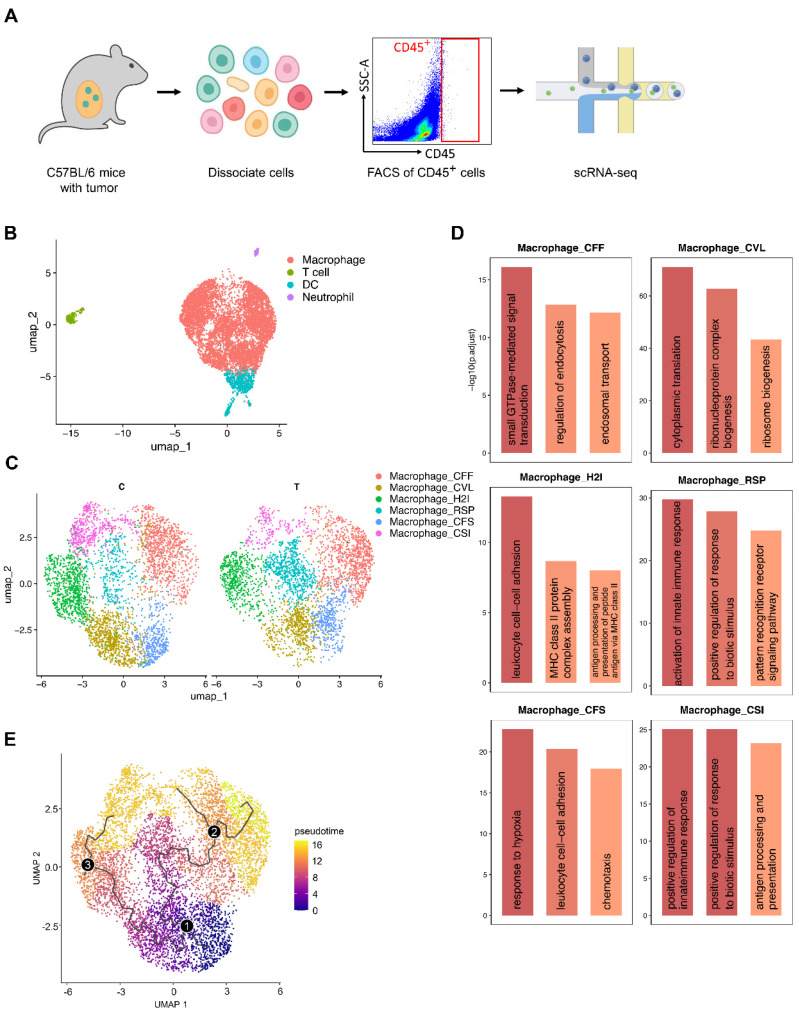
Macrophages dominate the tumor immune microenvironment and exhibit high heterogeneity. (**A**) Flow diagram of cell sorting and single-cell RNA sequencing (scRNA-seq). Cells within the harvested tumors were dissociated for flow cytometry. CD45^+^ immune cells were isolated by fluorescence-activated cell sorting (FACS) and used for scRNA-seq. (**B**) Uniform Manifold Approximation and Projection (UMAP) plot showing all identified cell types. (**C**) UMAP plot showing macrophage subclusters in the control (C) and the telmisartan-treated (T) group. (**D**) Top significantly enriched Gene Ontology (GO) pathways showing the functional characteristics of each macrophage subcluster. The y-axis represents the statistical significance unit of the GO enrichment, expressed as the −log10 (adjusted *p*-value). (**E**) UMAP visualization of pseudotime trajectory of macrophages inferred by Monocle3. Numbered circles represent principal points on the inferred trajectory graph, where point 1 indicates the root node (pseudotime origin), and points 2 and 3 denote distinct terminal states.

Given the established anti-proliferative effects of telmisartan on tumor cells [15,19,21], we hypothesized that its efficacy might involve modulating the tumor immune microenvironment. Therefore, we performed scRNA-seq on CD45^+^ immune cells isolated from both control (C) and telmisartan-treated (T) tumor tissues by fluorescence-activated cell sorting (FACS) to characterize the immune landscape (Figure 2A). Following stringent quality control, normalization, and scaling, four major cell types were defined (Figure 2B and Figure S4). Notably, macrophages accounted for over 70% of all immune cells, indicating a macrophage-dominated microenvironment. Furthermore, canonical marker analysis indicated that all identified macrophages were monocyte-derived rather than tissue-resident (Figure S5) [35,36,37]. Next, these macrophages were extracted and partitioned into six subclusters. For concise referencing, we assigned descriptive labels reflecting each cluster’s top three differentially expressed genes: macrophage_CFF (*Cbr2*^+^ *Fcrls*^+^ *Folr2*^+^), macrophage_CVL (*Cd24a*^high^ *Vcan*^high^ *Lilra6*^high^), macrophage_H2I (*H2-Eb1*^high^ *H2-Ab1*^high^ *Il1r2*^high^), macrophage_RSP (*Rsad2*^+^ *Slfn4*^+^ *Plac8*^+^), macrophage_CFS (*Cxcl3*^+^ *F3*^+^ *Slpi*^+^), and macrophage_CSI (*Cxcl9*^+^ *Serpina3g*^+^ *Iigp1*^+^) (Figure S6). Surprisingly, the distribution of these macrophage subclusters did not show a significant difference between the telmisartan-treated and control groups (Figure 2C), suggesting that telmisartan’s effect might be transcriptional rather than compositional. Functional annotation of enriched Gene Ontology (GO) terms revealed high heterogeneity among the macrophage subclusters: macrophage_CFF was associated with endosomal transport and phagocytosis, macrophage_CVL with ribosome biogenesis and active translation, macrophage_H2I with major histocompatibility complex (MHC) Class II assembly and antigen presentation, macrophage_RSP with immune response activation and pattern recognition, macrophage_CFS with response to hypoxia and chemotaxis, and macrophage_CSI with immune response and antigen presentation (Figure 2D). Finally, trajectory analysis was performed using Monocle3 and macrophage_CFS was selected as the putative root for pseudotime ordering because this subcluster displayed the highest chemotaxis and monocyte-associated signatures (Figure 2D and Figure S6). The analysis indicated that the macrophages differentiated from macrophage_CFS into more functionally distinct end-states, primarily observed in macrophage_CFF, macrophage_H2I, and macrophage_CSI (Figure 2E). This inferred developmental trajectory aligns with the distinct functional programs defined by GO analysis.

### 3.3. Telmisartan Downregulates Tumor-Promoting Signatures and Suppresses the Proliferation-Related Transcriptional Pathways in Macrophages

**Figure 3:**
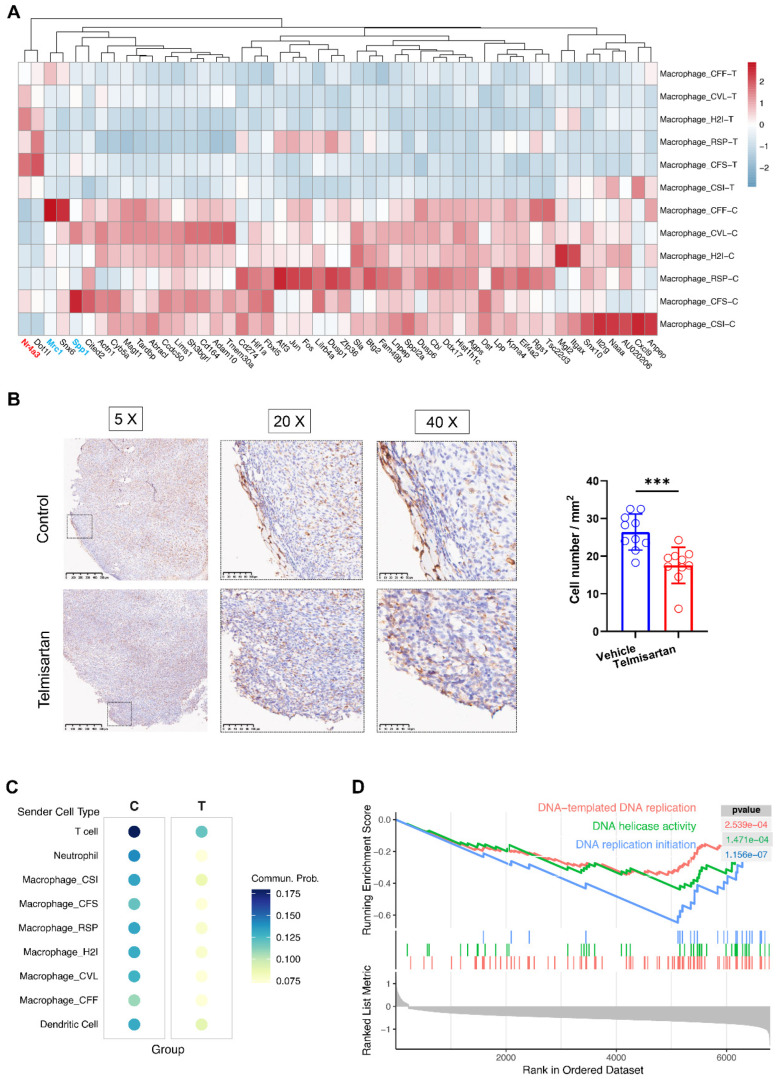
Telmisartan downregulates tumor-promoting signatures and suppresses the proliferation of macrophages. (**A**) Heatmap showing top 50 differentially expressed genes between the two groups across all macrophage subclusters. (**B**) Immunohistochemistry photos of CD206^+^ cells at the tumor border (n = 5/group). The difference in cell numbers between the two groups was analyzed using the *t*-test. (**C**) Dot plot showing the inferred communication probability of the *Ptprc-Mrc1* interaction in the two groups. Macrophage_CFF was selected as the target cells due to their prominent expression of *Mrc1*. (**D**) Gene set enrichment analysis (GSEA) revealed downregulated pathways linked to cell proliferation in the telmisartan-treated group. *** *p* < 0.001.

To further determine the effect of telmisartan on macrophages, we conducted differential gene expression analysis between the two groups across all subclusters (Figure 3A). Strikingly, the expressions of most genes were downregulated in the telmisartan-treated group, as only 4 out of the 601 genes that showed significant differential expression across all subgroups were upregulated. Among the downregulated genes were representative and widely reported pro-tumoral signature markers, such as the M2-like marker *Mrc1* and the osteopontin gene *Spp1* [38,39,40,41]. To validate this finding at the protein level, we performed immunohistochemistry (IHC) for CD206 (the protein product of *Mrc1*). Consistent with the sequencing data, CD206 expression was significantly reduced at the tumor border in the telmisartan-treated group (Figure 3B). However, this difference was not observed in the tumor core (Figure S7). Furthermore, we performed cell–cell communication analysis. The inferred communication probability of the *Ptprc-Mrc1* interaction between all cell types and macrophage_CFF (which exhibited the highest *Mrc1* expression) was markedly lower in the telmisartan-treated group (Figure 3C). Finally, Gene Set Enrichment Analysis (GSEA) revealed that pathways linked to cell proliferation were significantly downregulated in the telmisartan-treated group (Figure 3D). Together with the widespread gene downregulation, this indicates that telmisartan suppresses the overall activity and proliferative capacity of these tumor-associated macrophages.

### 3.4. Telmisartan Facilitates the Infiltration of Cytotoxic T Cells and Enhances MHC Class I Antigen Presentation

**Figure 4:**
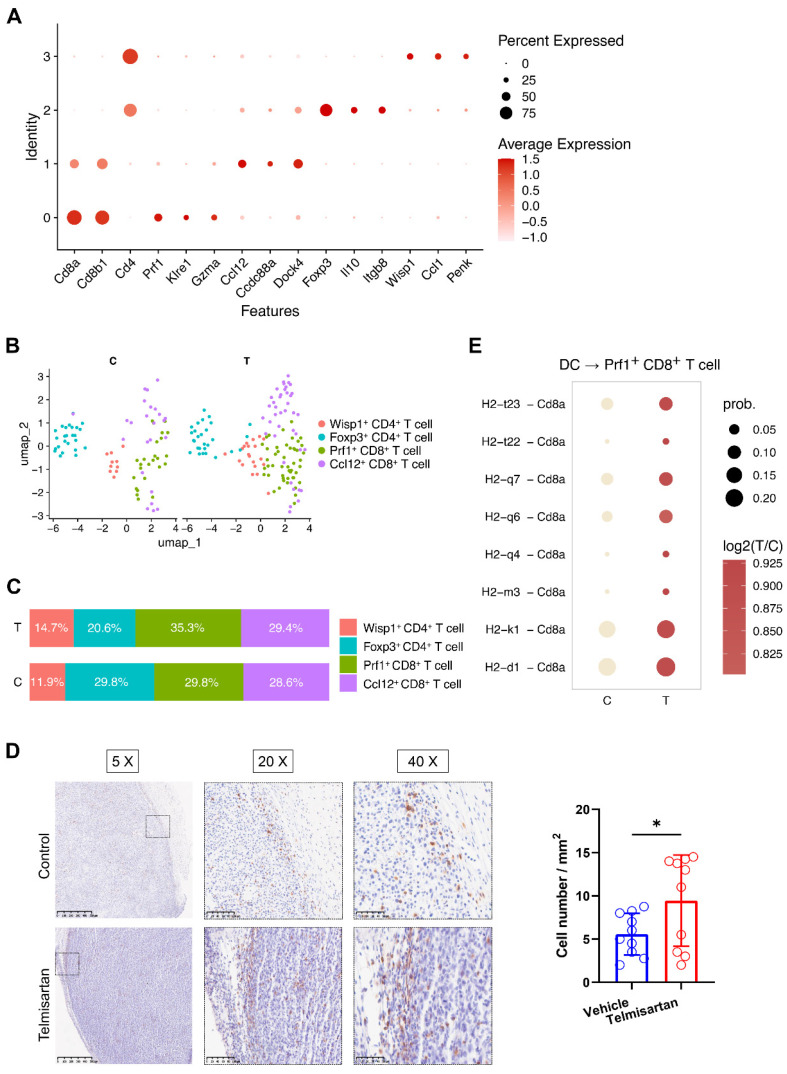
Telmisartan facilitates the infiltration of cytotoxic T cells and enhances major histocompatibility complex (MHC) class I antigen presentation. (**A**) Dot plot showing the top 3 markers of each T cell subcluster (two CD4^+^ subclusters and two CD8^+^ subclusters). (**B**) UMAP plot showing all identified T cell subclusters. (**C**) Stacked bar plots showing the proportion of T cell subclusters in the two groups. (**D**) Immunohistochemistry photos of CD8^+^ cells at the tumor border in the two groups (n = 5/group). The difference in cell numbers between the two groups was analyzed using the *t*-test. (**E**) Dot plot showing the inferred communication probability of MHC class I interactions between dendritic cells (DCs) and *Prf1*^+^ CD8^+^ T cells in the two groups. Cd8a was selected as a representation of MHC class I receptors. * *p* < 0.05.

To investigate the broader impact of telmisartan on the tumor immune microenvironment, we next turned our attention to T cells. Four T cells subclusters were identified, including two CD4^+^ and two CD8^+^ subclusters (Figure 4A,B). Specifically, the *Prf1*^+^ CD8^+^ T cell cluster expressed canonical cytotoxic markers (e.g., *Prf1* and *Gzma*), while the *Foxp3*^+^ CD4^+^ T cell cluster expressed classical regulatory T cell markers (e.g., *Foxp3* and *Il10*). Compositional analysis revealed a phenotypic shift in the T cell compartment following treatment. The telmisartan-treated group exhibited an increased proportion of *Prf1*^+^ cytotoxic T cells and a concomitant decrease in *Foxp3*^+^ regulatory T cells (Figure 4C). This finding was validated by IHC, which demonstrated a significant increase in the infiltration of CD8^+^ T cells at the tumor border in telmisartan-treated tumors (Figure 4D). However, similar to the findings for macrophages, this difference was absent in the tumor core (Figure S8). To explore the mechanism driving this cytotoxic T cell response, we investigated cell–cell communication. We found that inferred interactions between dendritic cells (DCs) or macrophages and *Prf1*^+^ cytotoxic T cells, specifically via MHC class I pathways, were substantially upregulated in the telmisartan-treated group (Figure 4E and Figure S9). This suggests that telmisartan may foster a more robust anti-tumor immune response by enhancing DC-mediated antigen presentation to cytotoxic T cells.

## 4. Discussion

Abundant macrophage infiltration is a well-recognized hallmark of the microenvironment of solid tumors, often comprising roughly half of the immune compartment [42], and this proportion was even higher in our model. While macrophages perform essential roles in host defense and immune homeostasis, tumor-associated macrophages (TAMs) frequently acquire immunosuppressive, tumor-promoting phenotypes [43,44]. Importantly, single-cell profiling studies have revealed a continuum of macrophage states that is not fully captured by the traditional binary M1-M2 polarization paradigm, calling that simple classification into question [45]. Nonetheless, canonical M2-associated markers such as CD206 (*Mrc1*) and CD163 remain correlated with adverse outcomes in diverse human cancers and therefore retain practical value for TAM characterization [46,47]. Guided by this rationale, we focused on differential *Mrc1* expression between groups as a tractable readout of telmisartan-associated macrophage changes.

Hypoxia is another defining feature of solid tumors and strongly influences macrophage recruitment and phenotype. Rapid tumor growth and vascular disruption create an imbalance between oxygen supply and demand, producing hypoxic niches that recruit macrophages via semaphorin 3A-neuropilin-1 signaling and retain them through plexinA1/plexinA4-mediated stop signals [48,49]. Hypoxic conditioning then drives macrophages toward tumor-promoting and pro-angiogenic functional programs [50]. This mechanism is reflected in our data: macrophage_CFS showed early migratory characteristics and was highly enriched for hypoxia-responsive pathways, and these hypoxia-associated macrophages expressed elevated levels of established TAM markers like *Spp1*.

Despite the limited exploration of telmisartan’s relationship with tumor immunity, its anti-inflammatory properties have been widely documented in inflammatory diseases. In rat models of chronic inflammation, telmisartan exhibits significant anti-inflammatory activity [51]. Chronic inflammation is closely associated with cancer development, particularly in CRC, where inflammatory bowel disease serves as a major risk factor [52]. Numerous oncogenic genes are capable of driving inflammatory responses, while proinflammatory cytokines promote tumor cell proliferation and angiogenesis [53]. Consequently, the tumor microenvironment resembles a chronic inflammatory state that facilitates tumor progression. TAMs play a central role in this process. Their infiltration reflects an inflammatory milieu and contributes to a tumor-promoting environment. Therefore, the overall suppressive effects of telmisartan on macrophages observed in our study likely reflect its anti-inflammatory action, which may also confer benefits in the context of tumor immunity. Intriguingly, despite the general trend toward transcriptional downregulation, the response was not entirely suppressive in the telmisartan-treated group. Among the few upregulated genes, we detected *Nr4a3* (Figure 3A), a transcription factor recently linked to M1-like, antitumor macrophage programs [54]. These findings suggest that telmisartan may induce heterogeneous and multifaceted effects on macrophage phenotypic reprogramming. Because TAMs can also promote tumor progression through angiogenesis, we evaluated vascular changes by IHC for CD31. Surprisingly, it did not reveal significant differences in vessel density or overt sprouting between groups (Figure S10), suggesting that telmisartan’s dominant immunomodulatory impact in our model is through macrophage transcriptional programs rather than alterations in endothelial architecture. However, this could also be attributed to an insufficient experimental duration, which may have precluded the observation of overt vascular effects.

Although macrophages constitute the predominant immune population within the tumor microenvironment, the contribution of T cells is equally crucial. As central mediators of adaptive immunity, T cells are key effectors in antitumor responses, with cytotoxic CD8^+^ T cells serving as primary executors of tumor cell killing. The T cell-enhancing potential of other ARBs such as candesartan has been reported as they increase the frequency of CD8^+^ T cells and decrease that of CD4^+^ Foxp3^+^ regulatory T cells [27]. This observation aligns with our findings that telmisartan enhanced the proportion of cytotoxic T cells and promoted MHC class I antigen presentation. Unlike the predominantly suppressive transcriptional patterns seen in macrophages, telmisartan appears to act on CD8^+^ T cells through distinct, activating mechanisms. Notably, ARBs have been tested as adjuvants to immune checkpoint blockade and have yielded encouraging preclinical outcomes [26,27,55]. These results raise the possibility that telmisartan could be repurposed as a combinatorial agent to potentiate immunotherapy in CRC. However, given the extensive macrophage infiltration and the comparatively low abundance of T cells in our dataset, the strength of our T cell-related conclusions remains limited and requires further validation through targeted experimental approaches.

Although this study reveals the immunomodulatory functions of telmisartan at the transcriptional level, investigations into its underlying mechanisms remain insufficient. As an AT1R blocker and a partial agonist of PPARγ, telmisartan may influence immunity through either or both of these pathways simultaneously. In chronic inflammatory diseases, AT1R signaling has been reported to promote T cell activation and macrophage M1 polarization [56,57]. While our findings appear to present a contrasting picture, other studies within the oncology context have similarly demonstrated that the inhibition of AT1R enhances the induction of tumor antigen-specific CD8^+^ cytotoxic T lymphocytes, suggesting that the precise role of AT1R blockers in tumor immunity warrants careful consideration. Furthermore, the role of PPARγ in tumor immunity is even more complex. In glioma, PPARγ activation can reduce IL-6 expression in astrocytes and exert antitumor functions [23]. Conversely, existing research indicates that the activation of PPARγ in macrophages leads to polarization toward tumor-associated M2 macrophages [58]. Therefore, further studies are necessary to delineate the precise pathways through which telmisartan regulates tumor immunity.

Several other findings in this study also warrant discussion. First, although calculating the tumor volume-to-body weight ratio partially mitigated the effects of body-weight differences, the telmisartan-treated group exhibited a 5% decrease in body weight at the experimental endpoint, in contrast to the 7% increase observed in the control group. This implies the potential presence of confounding factors such as drug toxicity and variations in general health status. Furthermore, because telmisartan administration commenced during the early stages of tumor inoculation—rather than after tumors were established and measurable—the extent to which its antitumor efficacy derives from direct intratumoral activity versus systemic effects remains to be elucidated. Second, significant differences in CD206 and CD8 expression were observed at the tumor border but not in the tumor core. Considering that the expression intensities of both markers were lower in the tumor core compared to the border, this may suggest a preferential accumulation of immune cells at the tumor periphery, reflecting limited penetrance into the central core. Finally, this study utilized a subcutaneous tumor model, which may exhibit distinct immune response patterns and pharmacological sensitivities compared to orthotopic tumor models.

## 5. Conclusions

In conclusion, our study demonstrates that the antihypertensive agent telmisartan exerts potent antitumor effects in a preclinical colorectal cancer model by profoundly reshaping the tumor immune microenvironment. Through high-resolution single-cell RNA sequencing, we revealed that telmisartan elicits a dual immunomodulatory response: it broadly attenuates the immunosuppressive and pro-tumoral transcriptional programs of tumor-associated macrophages while concurrently augmenting the infiltration and activation of cytotoxic CD8^+^ T cells via enhanced antigen presentation. Although the precise molecular mechanisms distinguishing its AT1R-blocking properties from its PPARγ-activating effects necessitate further investigation, our findings highlight the capacity of telmisartan to overcome immune evasion in the tumor periphery. Ultimately, this study provides a compelling preclinical framework for repurposing telmisartan—a safe, accessible, and highly tolerable drug—as a promising combinatorial agent to potentiate the efficacy of modern immunotherapies in colorectal cancer.

## Data Availability

The raw scRNA-seq data are available in the Gene Expression Omnibus database (accession number GSE311461). Other data used and/or analyzed in the current study are available from the corresponding authors upon reasonable request.

## Supplementary Materials

The following supporting information can be downloaded at: https://www.mdpi.com/article/10.3390/cells15080729/s1, Figure S1: Chemical structural formulas of telmisartan, valsartan, and candesartan; Figure S2: Dot plot showing the expression of canonical markers of non-macrophage clusters. Macrophage markers were excluded due to the potential confounding from abundant macrophage infiltration; Figure S3: Line graphs showing the variation trend of body weight of each group during the experiment. The significance of the main effect of the factors was measured using two-way repeated measures ANOVA. Statistical differences between the vehicle and telmisartan groups at specific individual time points were determined using a post hoc Šídák’s multiple comparisons test. * *p* < 0.05, ** *p* < 0.01; Figure S4: Violin plot showing the quality control data including the number of expressed genes (nFeature_RNA), the number of UMI counts (nCount_RNA), and the percentage of mitochondrial genes (percent.mt) in the two groups; Figure S5: Violin plot showing the expression of macrophage markers, monocyte-derived macrophage markers, and tissue-resident macrophage markers in the macrophage subclusters; Figure S6: Dot plot showing the top 3 markers of each macrophage subcluster; Figure S7: Immunohistochemistry photos of CD206^+^ cells at the tumor border (n = 5/group). The difference in cell numbers between the two groups was analyzed using the *t*-test; Figure S8: Immunohistochemistry photos of CD8^+^ cells at the tumor border (n = 5/group). The difference in cell numbers between the two groups was analyzed using the *t*-test; Figure S9: Relative communication probability between all macrophages and T cells in the two groups; Figure S10: Effect of telmisartan on tumor blood vessels. (A) IHC photo of CD31^+^ cells in the two groups. The red dash rectangular represents the tumor core, and the black dash rectangular represents the tumor border. (B) Histograms showing CD31^+^ blood vessels per mm^2^, sprouting vessels, direct distance between two ends, and vessel diameter of the two groups in tumor border. (C) Histograms showing CD31^+^ blood vessels per mm^2^, sprouting vessels, direct distance between two ends, and vessel diameter of the two groups in tumor core. *t*-test, * *p* < 0.05.

## Abbreviations

The following abbreviations are used in this manuscript:

CRC	colorectal cancer
Ang II	angiotensin II
AT1R	angiotansin II type 1 receptor
ARB	angiotensin II type 1 receptor blocker
PPARγ	peroxisome proliferator-activated receptor γ
scRNA-seq	single-cell RNA sequencing
i.g.	intragastric
FACS	fluorescence-activated cell sorting
GO	Gene Ontology
MHC	major histocompatibility complex
IHC	immunohistochemistry
GSEA	gene set enrichment analysis
DC	dendritic cell
TAM	tumor-associated macrophage
UMAP	uniform manifold approximation and projection
