# Supplementary figures and images for: Single-Cell Transcriptomics Reveals Immune Modulation by Telmisartan in Colorectal Cancer
Source: Cells. 2026 Apr 20;15(8):729. doi: 10.3390/cells15080729 (PMC13114542; doi:10.3390/cells15080729)

Telmisartan

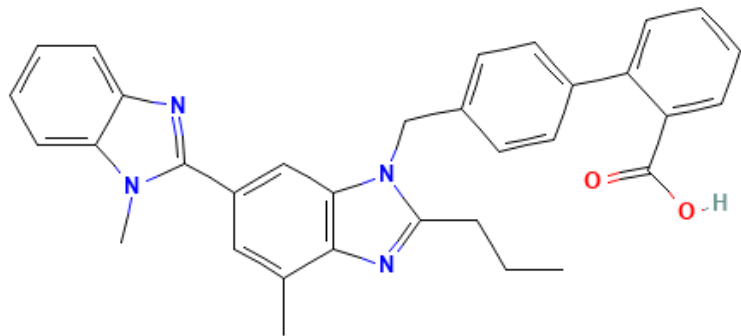

Valsartan

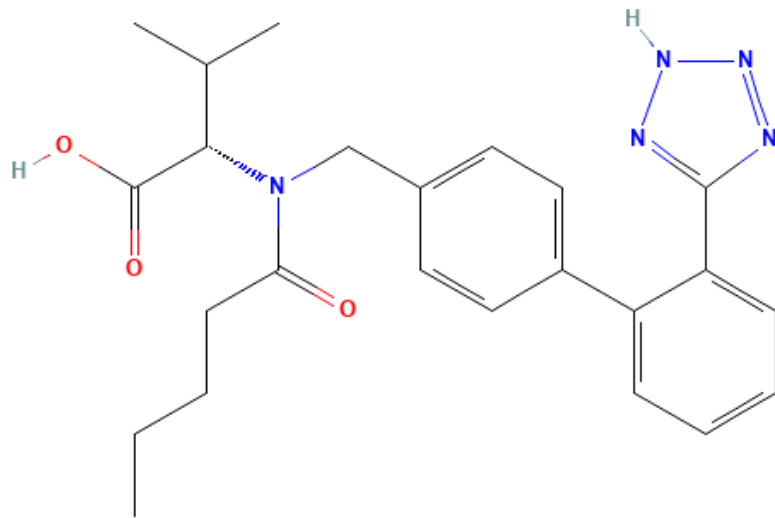

Candesartan

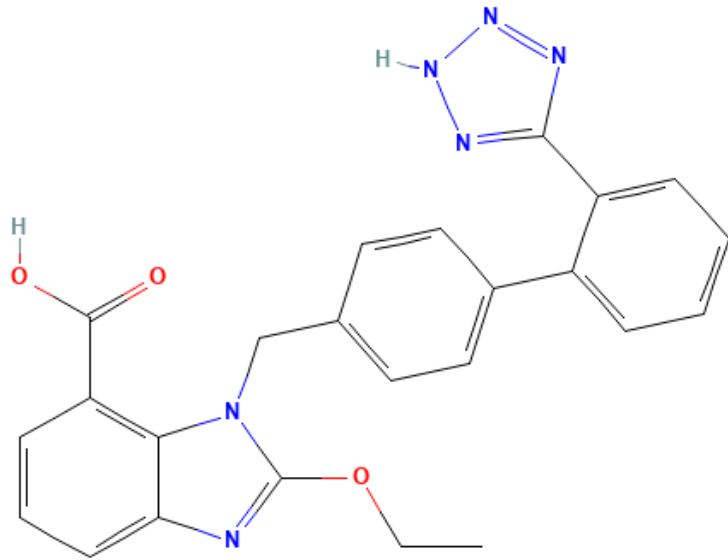

Supplement: Supplementary file 1 [file cells-15-00729-s001.zip › Fig. S1.pdf]

A

Control

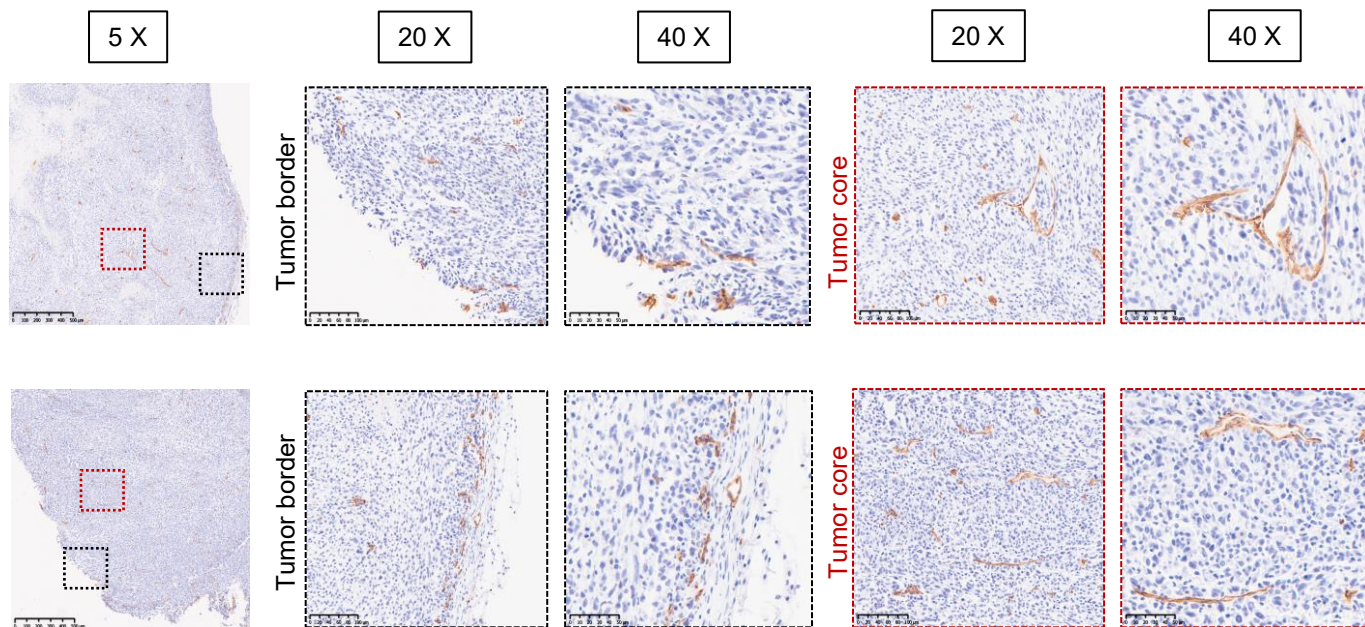

B

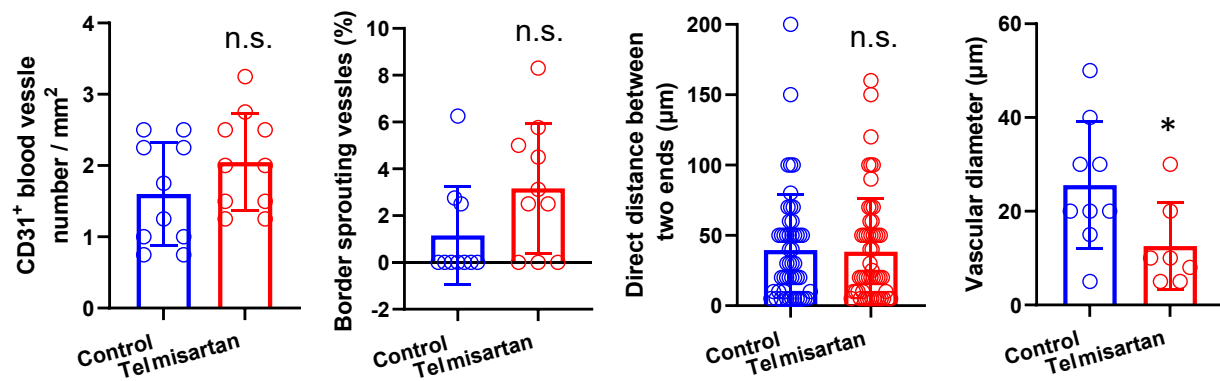

C

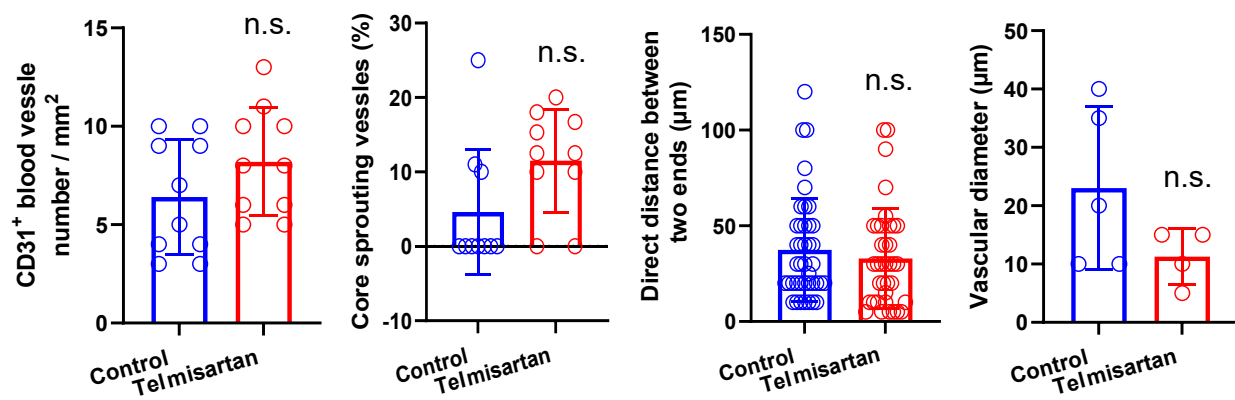

Supplement: Supplementary file 1 [file cells-15-00729-s001.zip › Fig. S10.pdf]

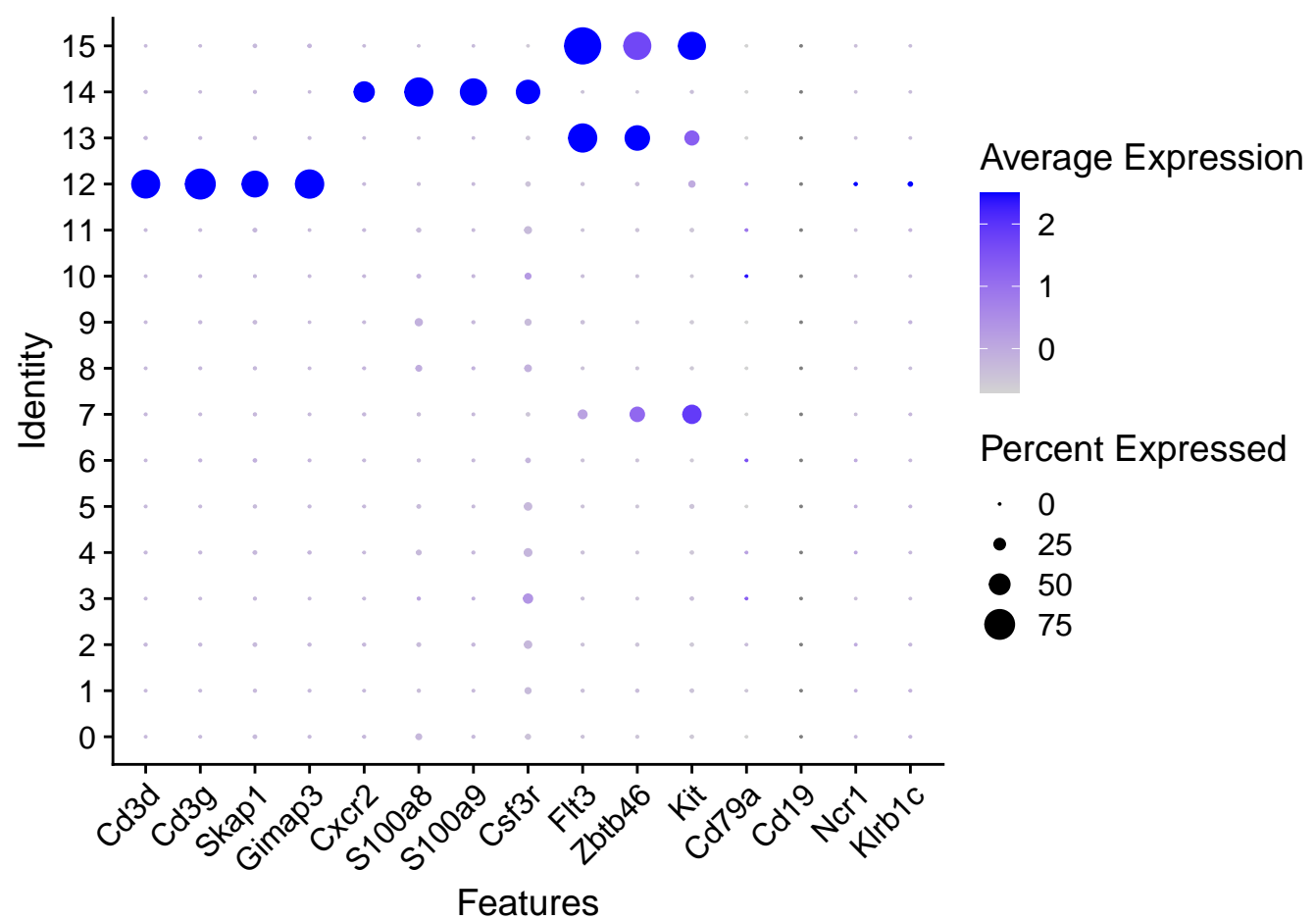

Supplement: Supplementary file 1 [file cells-15-00729-s001.zip › Fig. S2.pdf]

| Factor           | <i>P</i> value |
|------------------|----------------|
| Treatment        | 0.0009         |
| Time             | ns             |
| Treatment x time | 0.0008         |

● Vehicle  
● Telmisartan

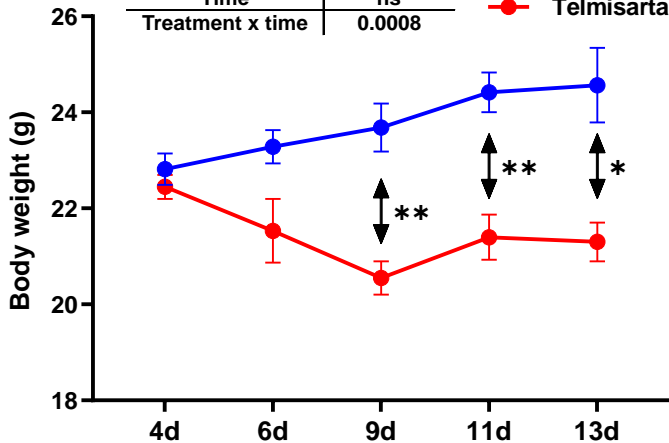

Supplement: Supplementary file 1 [file cells-15-00729-s001.zip › Fig. S3.pdf]

**nFeature\_RNA**

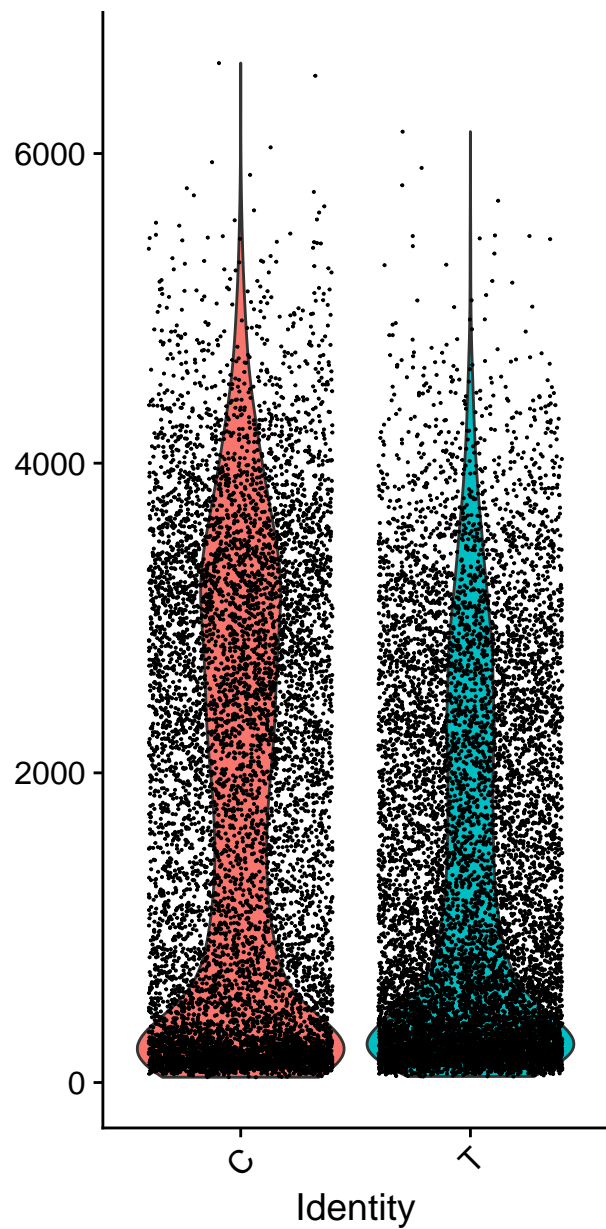

**nCount\_RNA**

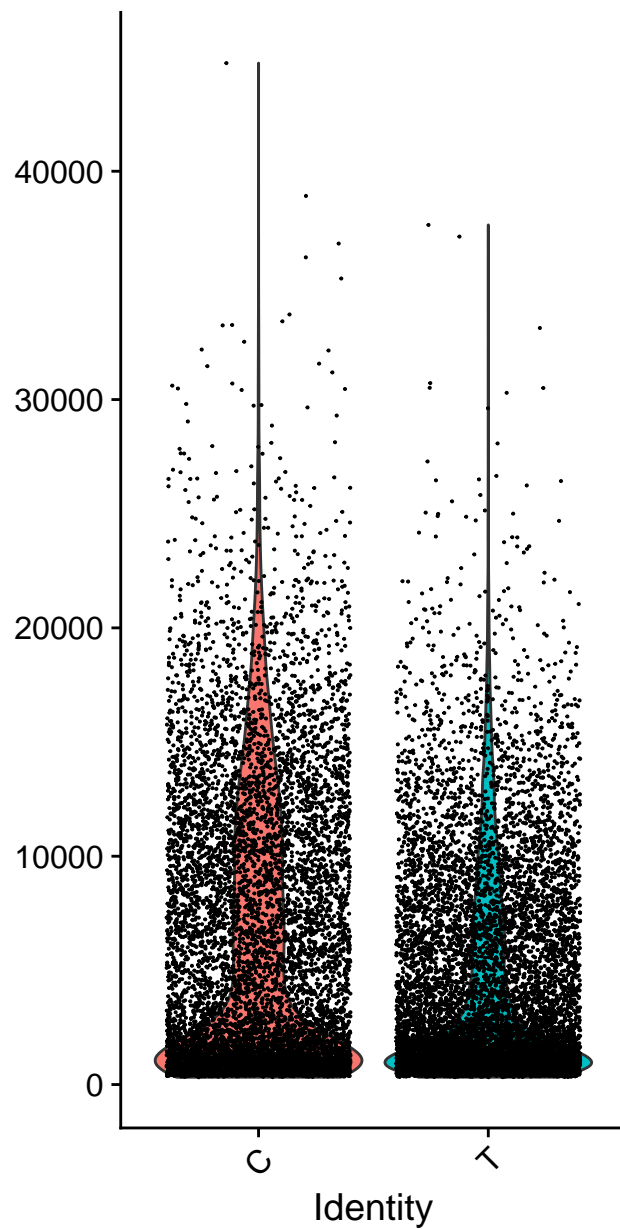

**percent.mt**

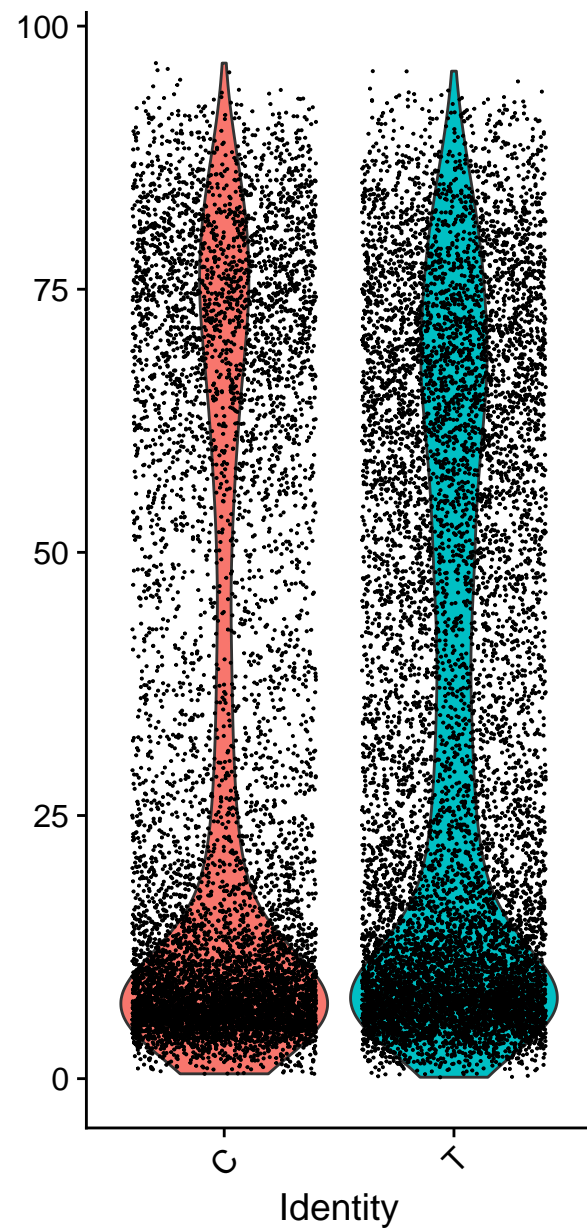

Supplement: Supplementary file 1 [file cells-15-00729-s001.zip › Fig. S4.pdf]

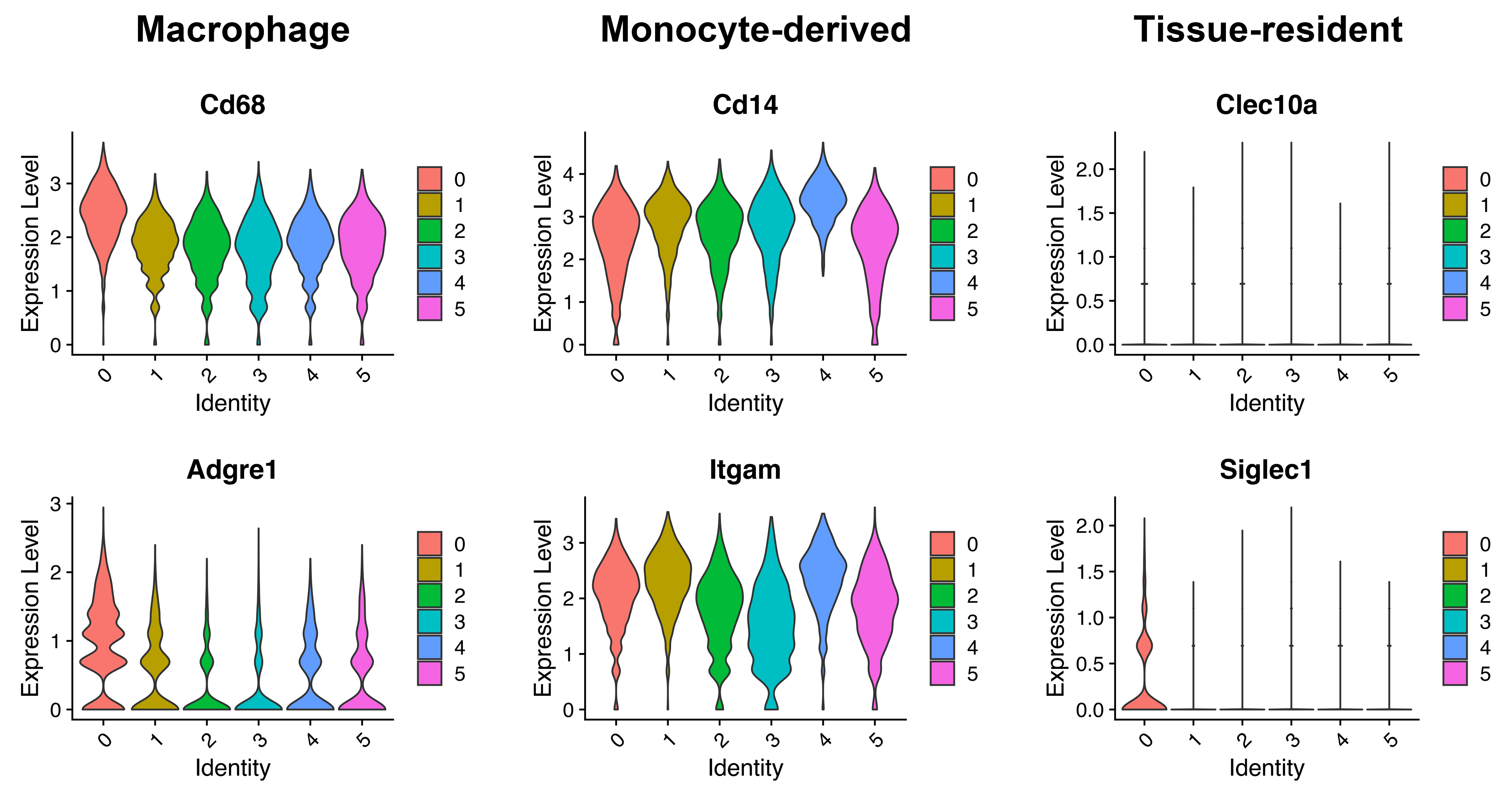

Supplement: Supplementary file 1 [file cells-15-00729-s001.zip › Fig. S5.png]

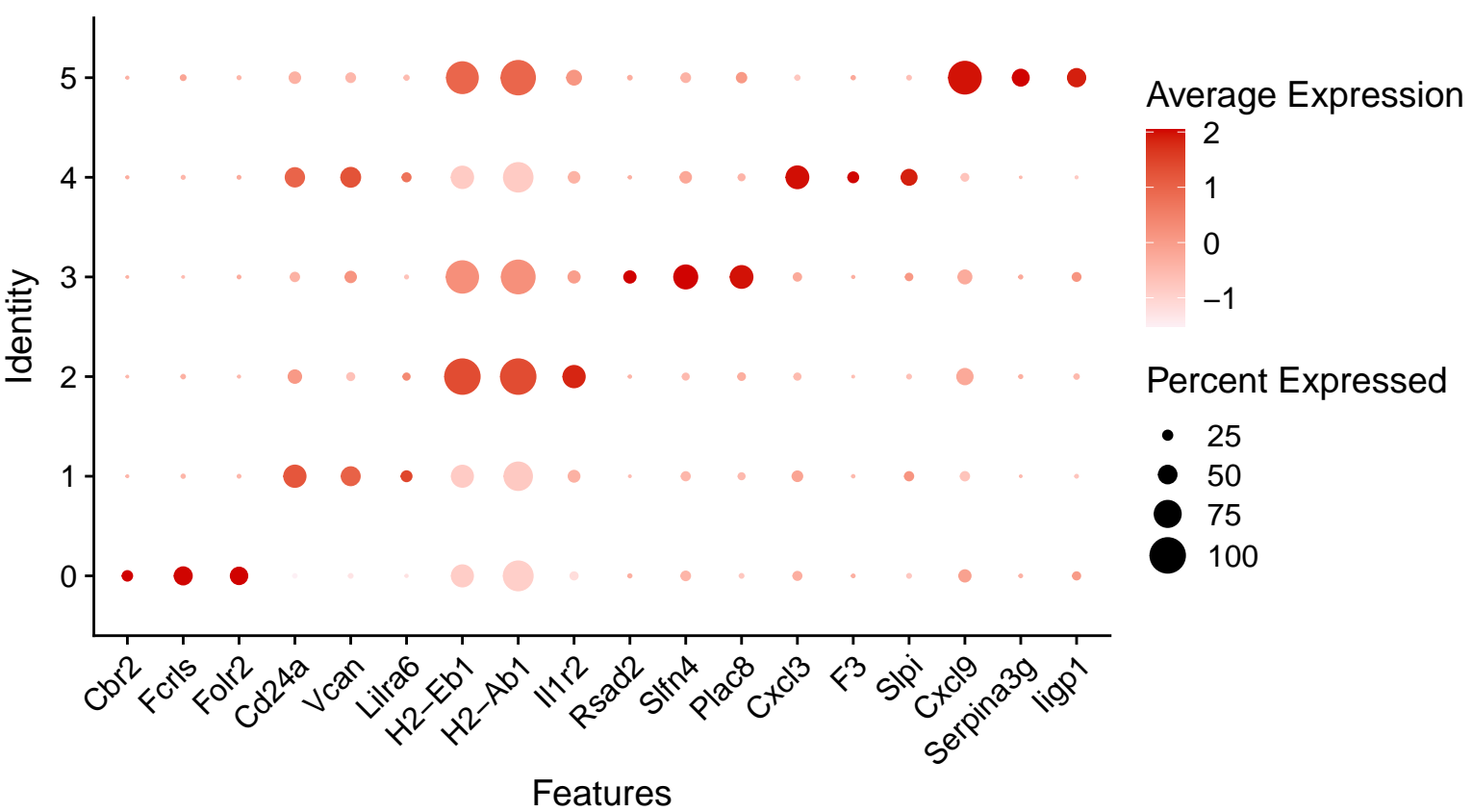

Supplement: Supplementary file 1 [file cells-15-00729-s001.zip › Fig. S6.pdf]

Control

5 X

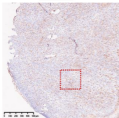

20 X

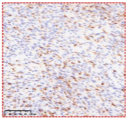

40 X

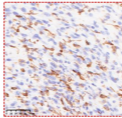

Telmisartan

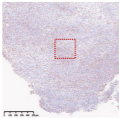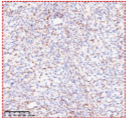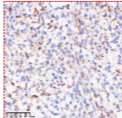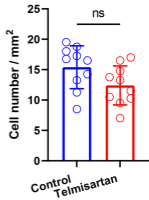

Supplement: Supplementary file 1 [file cells-15-00729-s001.zip › Fig. S7.pdf]

5 X

20 X

40 X

Control

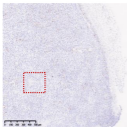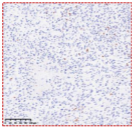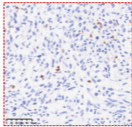

Telmisartan

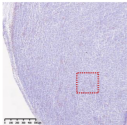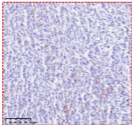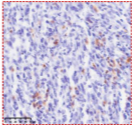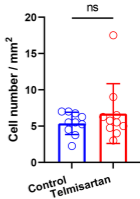

Supplement: Supplementary file 1 [file cells-15-00729-s001.zip › Fig. S8.pdf]

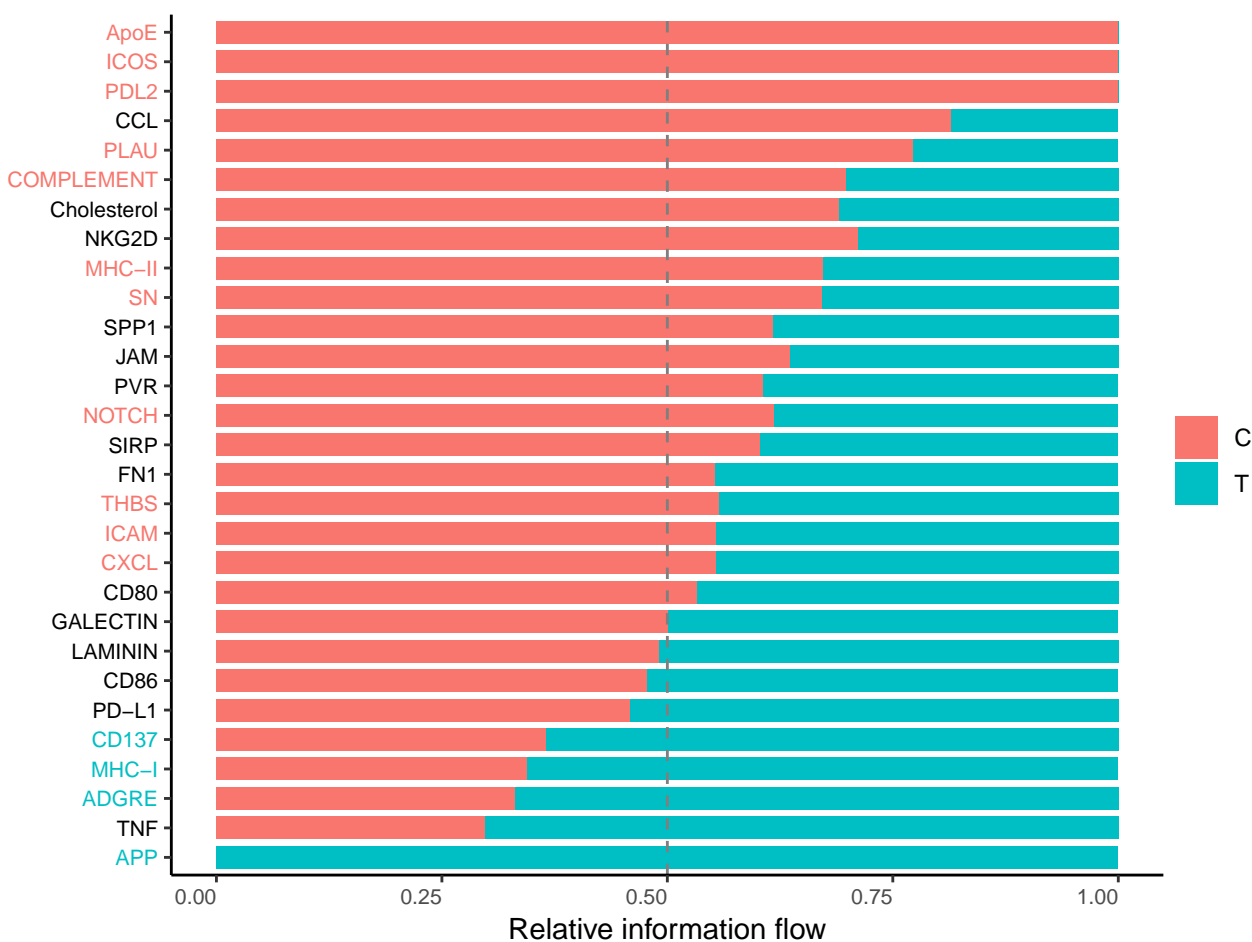

Supplement: Supplementary file 1 [file cells-15-00729-s001.zip › Fig. S9.pdf]
